# Multi-Target Antibacterial Mechanism of Moringin From *Moringa oleifera* Seeds Against *Listeria monocytogenes*

**DOI:** 10.3389/fmicb.2022.925291

**Published:** 2022-06-08

**Authors:** Yanlong Wen, Wenyun Li, Rongzhen Su, Min Yang, Nan Zhang, Ximing Li, Lingfei Li, Jun Sheng, Yang Tian

**Affiliations:** ^1^College of Food Science and Technology, Yunnan Agricultural University, Kunming, China; ^2^National Research and Development Professional Center for Moringa Processing Technology, Yunnan Agricultural University, Kunming, China; ^3^Engineering Research Center of Development and Utilization of Food and Drug Homologous Resources, Ministry of Education, Yunnan Agricultural University, Kunming, China; ^4^Yunnan Engineering Research Center of Drug and Food Homologous Functional Food, Yunnan Agricultural University, Kunming, China

**Keywords:** isothiocyanate, moringin, *Moringa oleifera* seeds, antibacterial activity, *Listeria monocytogenes*

## Abstract

Moringin [4-(α-L-rhamnosyloxy) benzyl isothiocyanate] is an isothiocyanate from *Moringa oleifera* seeds. It is the bioactivated form of the glucosinolate precursor glucomoringin with various health benefits. However, few studies have examined the antibacterial activity of moringin. This study aimed to investigate the antimicrobial activity and mechanism of moringin against *Listeria monocytogenes*. The minimum inhibitory concentration (MIC), and growth curves were used to evaluate the bacteriostatic effect of moringin against *L. monocytogenes*. Transcriptome analysis by RNA sequencing was performed to elucidate the underlying mechanism of moringin against *L. monocytogenes*. The transcriptome results were validated. The results showed that moringin inhibited the growth of *L. monocytogenes* with a MIC of 400 μM. RNA sequencing results showed that the differences in the expression of genes related to the cell wall and membrane biosynthesis, phosphotransferase system (PTS), oxidative stress, energy metabolism, and DNA binding were significantly affected. As with the transcriptome results, the results of the mechanism verification found that moringin damaged the integrity of the cell wall and cell membrane, stimulated oxidative stress, interfered with energy metabolism and DNA replication, and finally led to the death of *L. monocytogenes*. The present study provides evidence that moringin exhibits strong antimicrobial activity against *L. monocytogenes* and insight into its potential mechanism.

## Introduction

*Listeria monocytogenes*, a partly anaerobic gram-positive bacillus, is widely found in water, soil, and plants (Gasanov et al., 2005; Wu et al., 2021). *L. monocytogenes* can contaminate foods, such as milk (Hunt et al., 2017), meat products (Liu et al., 2019), and vegetables (Beuchat, 1996). It can grow during processing and transportation and survive low temperatures and harsh conditions (Low and Donachie, 1997; Zhao et al., 2020b). *L. monocytogenes* is a zoonotic bacterium that can cause meningitis, sepsis, gastroenteritis, and other diseases in humans and animals (Portnoy et al., 1992; Low and Donachie, 1997). From 2013 to 2017, a total of 211 cases of *L. monocytogenes* infection were reported by the China Foodborne Disease Surveillance Network, and the number is increasing year by year (Li et al., 2019b). Human listeriosis, which is caused by the pathogen *L. monocytogenes*, accounts for approximately 1,600 cases and 250 deaths per year in the United States (Scallan et al., 2011). Between 2017 and 2018, there were 978 listeriosis cases recorded in South Africa, resulting in 183 deaths (Vhiriri et al., 2020). In addition, listeriosis caused by *L. monocytogenes* is ranked by the World Health Organization (WHO) as one of the deadliest food-borne diseases, and it has a 20–30% mortality rate (Mpondo et al., 2021), making it severe public health concern posing a great threat to human health.

Generally, antibiotics or chemical preservatives are used to inhibit *L. monocytogenes*. However, the overuse and misuse of antibiotics and chemical preservatives may cause adverse side effects on humans that are more harmful than the microbes themselves (Tian et al., 2012; Ordoñez et al., 2022). The emergence of safety risks from antibiotic-resistant bacteria and the risks of using chemical preservatives have both received increasing attention. Currently, thermal sterilization is the primary technical means of ensuring food safety. However, excessive heating can destroy heat-sensitive nutrients in foods (Mangundayao and Yasurin, 2017). Therefore, research on natural and safe antibacterial substances has become a priority.

Isothiocyanates (ITCs) are a class of sulfur-containing organic compound widely found in cruciferous plants that have biological activities, such as antibacterial (Romeo et al., 2018a,b), antioxidant, and anti-inflammatory effects (Burčul et al., 2018). Some isothiocyanates, such as allyl isothiocyanate, benzyl isothiocyanate, phenethyl isothiocyanate, and sulforaphane, have been shown to possess substantial chemo-preventative activity against various human malignancies (Singh and Singh, 2012). Moringin is a structurally unique isothiocyanate with an additional sugar residue in the side chain. *Moringa* seeds are a better source of moringin and contain glucomoringin approximately 8–10% (Mathiron et al., 2018). Studies have shown that moringin has anti-inflammatory (Cheenpracha et al., 2010), hypotensive (Faizi et al., 1994), and anticancer (Giacoppo et al., 2017) activities. However, little has been reported on the antibacterial activity of moringin and its inhibitory mechanism.

In the present study, the antibacterial activity of moringin against *L. monocytogenes* was evaluated by inhibition zones, minimum inhibitory concentration (MIC), and growth curves, and the differential gene expression was analyzed by transcriptomic technology. Furthermore, the effects of moringin on the cell morphology, cell membrane integrity, oxidative stress, energy metabolism, and DNA of *L. monocytogenes* were investigated to elucidate the potential mechanism by which moringin inhibits *L. monocytogenes*.

## Materials and Methods

### Bacterial Strain and Culture Conditions

The *L. monocytogenes* NDKM-NR-2019-034 used in the present study was isolated from bovine milk by our research group and identified using morphological and molecular biological methods. The strain was inoculated into the nutrient broth (NB) and cultured at 37°C for 18 h to the logarithmic growth phase. Moringin was obtained from *Moringa* seeds with reference to a previous method (Xie et al., 2021).

### Antimicrobial Activity of Moringin

#### Minimum Inhibitory Concentration

The antimicrobial effect of moringin against *L. monocytogenes* was examined *via* the broth microdilution method (Moreira et al., 2005). The suspension of *L. monocytogenes* (10^6^ CFU/ml) was incubated with 50–400 μΜ of moringin in nutrient broth. The minimum concentration of added moringin that inhibits visible bacterial growth is defined as the MIC.

#### Growth Curves

Growth curves were constructed according to the previously described method (Shi et al., 2016). The concentration of the bacterial solution was precisely adjusted to 10^6^ CFU/ml, and 10 μl of inoculum solution was added to each well of the 96-well plate. An equal volume (240 μl) of NB containing different concentrations of moringin was added to each well to obtain the final concentrations of 1/2 MIC, 1 MIC, and 2 MIC, and the inoculum solution without moringin was used as the control. All plates were incubated at 37°C. Cell growth was monitored every 3 h by measuring the optical density at 600 nm with a BioTek microplate reader (Molecular Device, United States).

### Transcriptomic Analysis

#### RNA-Seq and Bioinformatics Analysis

*L. monocytogenes* were treated with a 1/2 MIC concentration of moringin. Transcriptome sequencing analysis was performed on four treated samples and four control samples. The OD_260_/OD_280_ ratio of the RNA was determined using a Nanodrop 2000 (Thermo Fisher, United States). Prior to library formation, rRNA was removed using the Ribo-Zero rRNA Removal Kit (Epicentre, San Diego, CA, United States), and mRNAs were split into ~200 bp pieces using metal ions. The mRNA fragments were reverse transcribed into first-strand cDNA, followed by second-strand cDNA synthesis. Then, the UNG enzyme was used to digest the second-strand cDNA to generate libraries. Quality control was performed on the sequencing data, and low-quality reads were eliminated. Raw reads were filtered to generate clean reads and then submitted to bioinformatics analysis. The resulting high-quality reads were mapped onto the *L. monocytogenes* reference genome (accession No. GCA_004771175.1). The transcriptome sequencing data were analyzed using the free online platform of Majorbio Cloud Platform.^1^

#### Quantitative Real-Time PCR Validation

To assess the accuracy of the transcriptome sequencing analysis, qRT-PCR validation was performed using the same RNA samples as were used for RNA-Seq. Some differentially expressed genes were chosen from the transcriptome analysis, and the relative expression levels of these genes were determined using the 2^−ΔΔ*C*T^ method after normalization, with 16S rRNA serving as the internal reference gene. The primer sequences used in this study are shown in Table 1.

**Table 1:** The primers used for qRT-PCR in this study.

Target gene	Primer	Sequence (5′–3′)
16SrRNA	16SrRNA-F	GCGGCCCCCTGGACAATGAC
	16SrRNA-R	TAGCTAAGGAAGCCACGCCT
FORC67_2473	FORC67_2473-F	CTGCTGCGATTACTGGTGGA
	FORC67_2473-R	ACGTTCCACATCAGCACCAA
FORC67_1632	FORC67_1632-F	GGTACTGGAAGCGGGACAAA
	FORC67_1632-R	TGGATGGTGCGCGTAATCAT
FORC67_1456	FORC67_1456-F	CAGGACTTGGAGGCAGTTCC
	FORC67_1456-R	TAACCAAGCGCCACATCCAT
FORC67_1457	FORC67_1457-F	CGGCCATAACTTGCTTGTTCA
	FORC67_1457-R	AACAGGAATTGCCCCACCAA
FORC67_1458	FORC67_1458-F	TAAACTGGCGGACCGTATCG
	FORC67_1458-R	CGCATCTTCTACCGTACCCC
FORC67_0722	FORC67_0722-F	ACGATGCAATGTTCATGCGT
	FORC67_0722-R	CTCTAAAACCCGGTCCGCTT
FORC67_0533	FORC67_0533-F	AAGTGCGTGCGGTACTAGAC
	FORC67_0533-R	TCGCGAGTATTTCATCGATTGT

### The Effect of Moringin on the Cell Wall and Cell Membrane of *Listeria monocytogenes*

#### Scanning Electron Microscopy Observation

The morphological changes of *L. monocytogenes* after treatment with moringin were observed by SEM. Bacterial cells were cultured with media containing 1/2 MIC and 1 MIC concentrations of moringin, respectively, without moringin as a control. Treated and non-treated bacteria were incubated at 37°C for 24 h. After incubation, cells were collected by centrifugation at 6,000 *g* for 10 min, washed three times with PBS buffer, and then incubated with 2.5% glutaraldehyde in PBS at 4°C overnight. Following three washes with PBS, samples were treated with ascending concentrations of ethanol (30, 50, 70, 80, 90, and 100%). The samples were vacuum freeze-dried, fixed on an SEM mount, sputter-coated with gold under a vacuum, and observed by FlexSEM 1000 SEM (Hitachi High-Technologies, Tokyo, Japan).

#### Membrane Integrity Assay

The effect of moringin on the cell membrane integrity of *L. monocytogenes* was detected by propidium iodide (PI) staining according to a previously reported method (Tang et al., 2010). Bacterial suspensions treated with different concentrations of moringin were incubated at 37°C for 24 h and then fixed with PI (final concentration of 10 μg/ml) for 20 min at 4°C in the dark. After that, bacterial cells were collected by centrifugation at 6,000 *g* for 10 min, washed with 0.85% NaCl solution, and resuspended in the same saline to reach a concentration of 1 × 10^6^ CFU/ml before analysis. PI staining of the cells was observed with an inverted fluorescence microscope (Leica DMI 3000B, Germany).

### Effect of Moringin on Oxidative Stress in *Listeria monocytogenes*

#### Determination of Intracellular Malondialdehyde

Malondialdehyde (MDA) was determined by the microscale MDA assay kit (Nanjing Jiancheng Bioengineering Institute, Nanjing, China) according to a previously described protocol (Kim et al., 2020). *L. monocytogenes* cells were collected by centrifugation at 6,000 rpm for 10 min (10^6^ CFU/ml) and then washed twice with PBS (0.1 M, pH 7.4). Cells treated with different concentrations of moringin (0, 1/2 MIC, 1 MIC, and 2 MIC) were resuspended in PBS and sonicated on ice. The remaining debris was removed by centrifugation at 15,000 rpm at 4°C for 10 min. The supernatant was taken for MDA measurement according to the MDA assay kit’s instructions. The protein content was measured using a total protein quantitative assay kit (Nanjing Jiancheng Bioengineering Institute, Nanjing, China), using bovine serum albumin as the standard.

#### Detection of ROS Activity

Intracellular ROS levels were measured using the Reactive Oxygen Species Assay Kit (Nanjing Jiancheng Bioengineering Institute, Nanjing, China) according to the instructions. Briefly, cells treated with different concentrations of moringin (0, 1/2 MIC, 1 MIC, and 2 MIC) were incubated with 20 μM dichlorodihydrofluorescein diacetate (DCFH-DA) in Hanks’ balanced salt buffer for 30 min at 37°C. The ROS levels were measured using a fluorescence microplate reader (BioTek, HIM, United States) with an excitation wavelength of 485 nm and an emission wavelength of 525 nm.

### Molecular Docking Simulation

Molecular docking was performed using AutoDock Vina software (Trott and Olson, 2010). The crystal structure of DNA was obtained from the RCSB Protein Data Bank^2^ (PDB entry: 453D). The 3D structure of moringin was retrieved from PubChem.^3^ The input files were prepared by the AutodockTools 1.5.6 package (Sanner, 1999; Morris et al., 2009). Briefly, all water molecules and ligands inside the DNA model were removed, polar hydrogen atoms were added, and the Gasteiger charge was calculated. The binding sites were determined from the model’s intrinsic ligand (center_x = 15.117, center_y = 15.117, center_z = 8.718, size_x = 58, size_y = 56, size_z = 52). The grid box determination was conducted with a spacing of 0.375 Å. The final confirmation was selected according to the output binding affinity and calculated by Autodock Vina using its intrinsic AMBER forcefield scoring function. Interactions were analyzed by Ligplot^+^ software (Laskowski and Swindells, 2011) and visualized by PyMOL software.^4^

### Effect of Moringin on Energy Metabolism in *Listeria monocytogenes*

#### Determination of Intracellular ATP

ATP was determined according to a modified method referenced elsewhere (Zou et al., 2015). Intracellular ATP was detected using an ATP assay kit (Nanjing Jiancheng Bioengineering Institute, Nanjing, China). Cell suspensions (10^6^ CFU/ml) were treated with four concentrations of moringin (0, 1/2 MIC, 1 MIC, and 2 MIC). Briefly, 100 μl of the enzyme working solution was added to each well of the 96-well plate, mixed thoroughly, and kept at room temperature for 5 min. Then, an *L. monocytogenes* solution containing 20 μl of ATP lysate was transferred to each well of a 96-well plate (containing the enzyme working solution) and mixed quickly with a pipette. After an interval of at least 2 s, the ATP levels were measured using a BioTek microplate reader (Molecular Device, United States).

#### Measurement of the ATPase and Activity

The concentration of the *L. monocytogenes* solution was adjusted to 10^6^ CFU/ml, washed three times with PBS, and then centrifuged at 3,000 *g* for 10 min, followed by resuspension in PBS. Different concentrations of moringin were added to make the final concentrations of 1/2 MIC, 1 MIC, and 2 MIC, with the moringin-free inoculum solution used as a control. The ATPase activity was measured as described previously with slight modifications according to the manufacturer’s instructions (Nanjing Jiancheng Bioengineering Institute, Nanjing, China; Zhan et al., 2011).

### Statistical Analysis

The statistical analysis was conducted using GraphPad Prism 5.0 software. Comparisons between two groups were made using an independent *t*-test (two-tailed method), with a significance level of *p* < 0.05.

## Results

### Antimicrobial Activity of Moringin Against *Listeria monocytogenes*

#### MIC of Moringin Against *Listeria monocytogenes*

The inhibitory results of different concentrations of moringin on *L. monocytogenes* are presented in Table 2. After 24 h of incubation, no colony growth was observed in the moringin-containing plates when the mass concentration of moringin was 400 μM, indicating that the MIC of moringin on *L. monocytogenes* was 400 μM.

**Table 2:** Effects of different concentrations of moringin on *Listeria monocytogenes*.

Incubation (time/h)	Concentration of MITC/(μM)					
	0	50	100	200	400	800
24	+	+	+	+	−	−

*+ indicated significant colonies. − indicated significant no obvious colonies growth*.

#### Growth Curves of Moringin on *Listeria monocytogenes*

**Figure 1:**
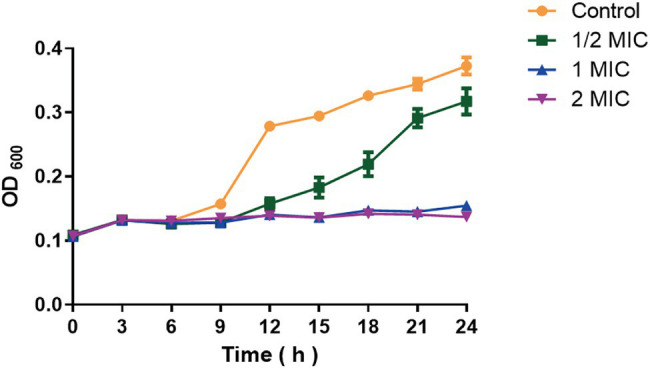
Growth curve of moringin on *Listeria monocytogenes*.

Different concentrations of moringin were used to treat *L. monocytogenes* to further investigate the inhibitory effect of moringin against *L. monocytogenes* (Figure 1). The results showed that 1 MIC and 2 MIC concentrations of moringin could completely inhibit the growth of *L. monocytogenes*, and 1/2 MIC also showed a certain inhibitory effect.

### Transcriptomic Changes in *Listeria monocytogenes* After Treatment With Moringin

#### Analysis of the Differentially Expressed Genes

**Figure 2:**
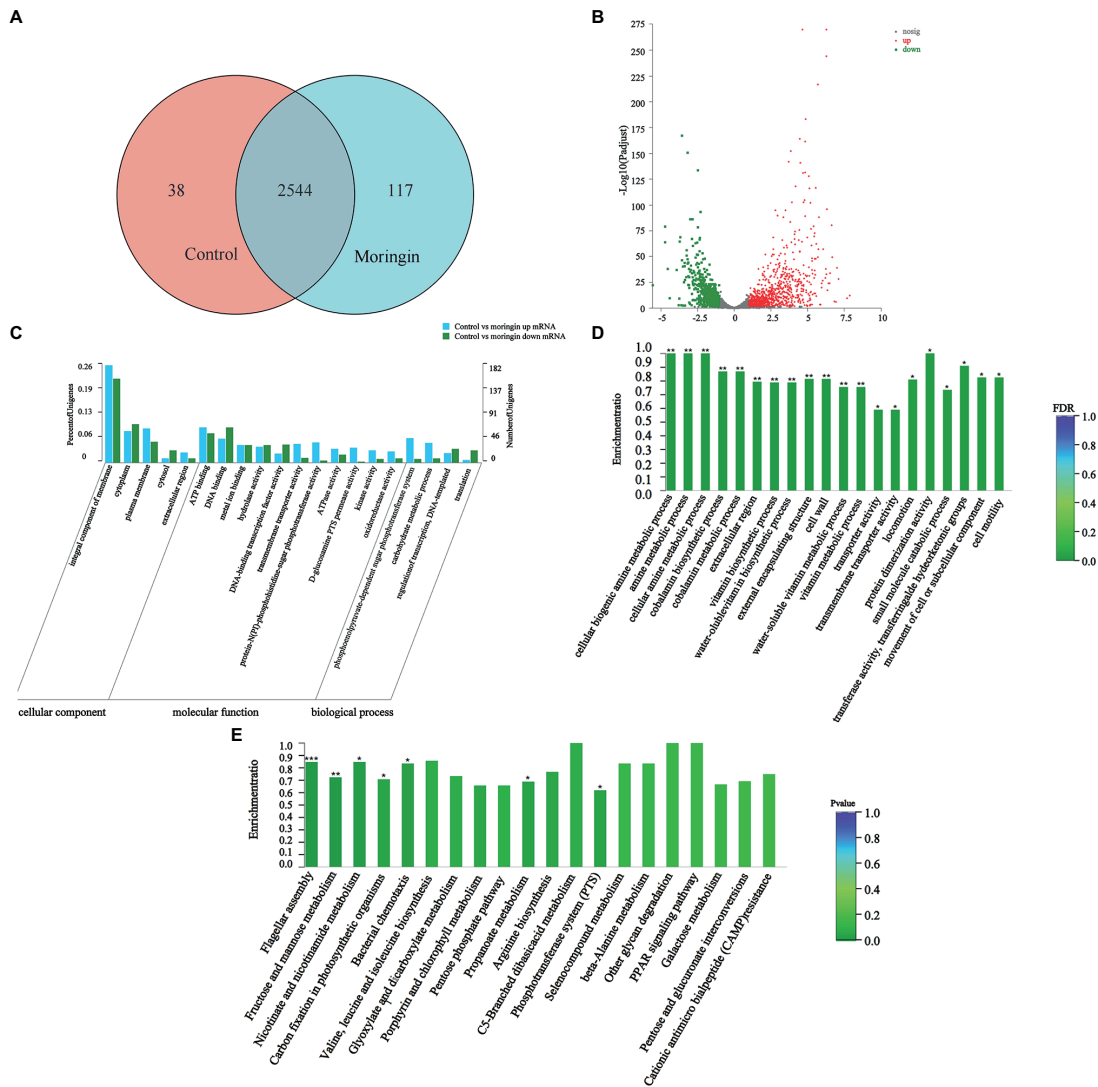
Transcriptome analyses of *Listeria monocytogenes* after treatment with moringin. **(A)** Venn diagram. **(B)** Percentages of up- and downregulated genes for each group indicated on a volcano plot. **(C)** GO classification of DEGs. **(D)** GO enrichment of DEGs. **(E)** Statistical enrichment of differentially expressed genes in KEGG pathways. ^*^p < 0.05, ^**^p < 0.01, and ^***^p < 0.001.

A total of 2,699 genes were detected in *L. monocytogenes* in the control and the moringin-treated groups. Among them, 38 and 117 differentially expressed genes (DEGs) were unique to the control and the moringin-treated groups, respectively (Figure 2A). A total of 794 genes were upregulated, and 606 genes were downregulated (Figure 2B).

#### Gene Ontology Analysis

Figure 2C shows the gene ontology (GO) categorization of the up- and downregulated genes in the treated cells. For the cellular component categories, the majority of GO terms were related to the integral component of the membrane. For molecular function categories, most GO terms were associated with ATP binding and DNA binding. For biological process categories, most GO terms were related to the phosphoenolpyruvate-dependent sugar phosphotransferase system.

The enrichment of GO groups was evaluated to better visualize the findings (Figure 2D). Many genes involved in cellular components were altered, such as cell wall (GO:0005618), external encapsulating structure (GO:0030312), and extracellular region (GO:0005576). This suggested that exposure to moringin caused changes in cell structure and cellular components. In addition, the expression levels of biological process genes were significantly different in *L. monocytogenes* after moringin treatment, especially in the cellular biogenic amine metabolic process (GO:0006576), amine metabolic process (GO:0009308), and cellular amine metabolic process (GO:0044106), indicating that moringin affected the metabolism of *L. monocytogenes*.

#### Kyoto Encyclopedia of Genes and Genomes Analysis

Finding alterations in biological functions is crucial in determining how antimicrobial activity works. The biological roles of genes were studied at the molecular, cellular, and organism levels using Kyoto Encyclopedia of Genes and Genomes (KEGG) pathway enrichment analysis (Figure 2E). In this study, seven pathways were found to be significantly enriched (*p* < 0.05) in *L. monocytogenes* after moringin treatment, including one from environmental information processing pathways, two from cellular processing pathways, and four from metabolism pathways. Nearly 57% of the pathways with significant alterations were associated with metabolism, suggesting that the mechanism by which *L. monocytogenes* was inhibited by moringin may involve metabolic changes in cells.

Additionally, a KEGG pathway analysis showed that moringin treatment affected peptidoglycan biosynthesis related to the *L. monocytogenes* cell wall structure and significantly downregulated the MurA and MurC genes (Supplementary Figure S1); significantly upregulated the PotA and PotD genes related to cell membrane integrity in the ABC transport system and significantly downregulated the opuA, opuB, and opuC genes (Supplementary Figure S2); significantly upregulated the FliH and FliI, FlgL and FlgK, CheA and Che genes related to cell movement (Supplementary Figures S3, S4); significantly upregulated phosphotransferase system MltA, GatA, GatB, and GatC genes (Supplementary Figure S5); and all genes of the tricarboxylic acid cycle related to energy metabolism were significantly downregulated (Supplementary Figure S6).

#### qRT-PCR Validation

**Figure 3:**
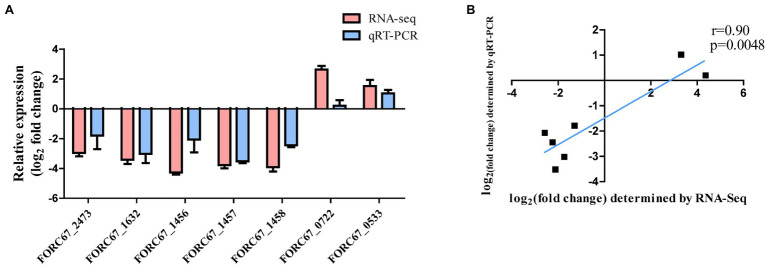
Comparison of RNA-Seq and qRT-PCR results. **(A)** Gene expression levels of RNA-Seq and qRT-PCR analysis. **(B)** Gene expression correlation analysis.

Seven DEGs (given in Table 1) were chosen for qRT-PCR assays to confirm the reliability of the RNA-Seq data. The results showed that the variation in trends of these seven genes based on qRT-PCR was consistent with those obtained by transcriptome sequencing (Figure 3A). The expression levels of the seven genes obtained by the transcriptomic analysis were positively correlated with those obtained by qRT-PCR (*r* = 0.90, *p* = 0.0048; Figure 3B).

### Validation of Antibacterial Mechanisms

#### Moringin Disrupted the Integrity of the Cell Wall and Cell Membrane of *Listeria monocytogenes*

**Figure 4:**
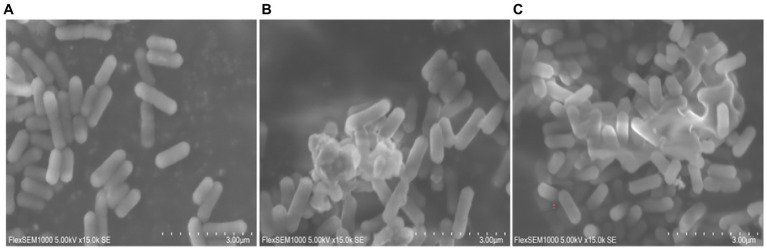
Effect of moringin on the surface structure and morphology of *Listeria monocytogenes* by SEM. **(A)** Untreated *Listeria monocytogenes*. **(B)** *Listeria monocytogenes* treated with moringin at 1/2 MIC. **(C)** *Listeria monocytogenes* treated with moringin at 1 MIC.

The cell membrane or cell wall is the integral component of all bacterial cells and is critical for bacterial protection. Once the outer membrane or cell wall of the bacteria is destroyed by physical or chemical action, the components of the cytoplasm leak out, resulting in bacteriostatic and bactericidal actions. Since the results of transcriptome sequencing showed that many genes involved in cellular components were altered after moringin treatment, the surface structure and morphology of *L. monocytogenes* were investigated by SEM. As shown in Figure 4, normal *L. monocytogenes* showed a rod-shaped, intact, round, and smooth surface. In contrast, after moringin treatment, the cell surface of *L. monocytogenes* was disrupted, the cells appeared wrinkled, the contents of cells leaked out, and many cells adhered together.

**Figure 5:**
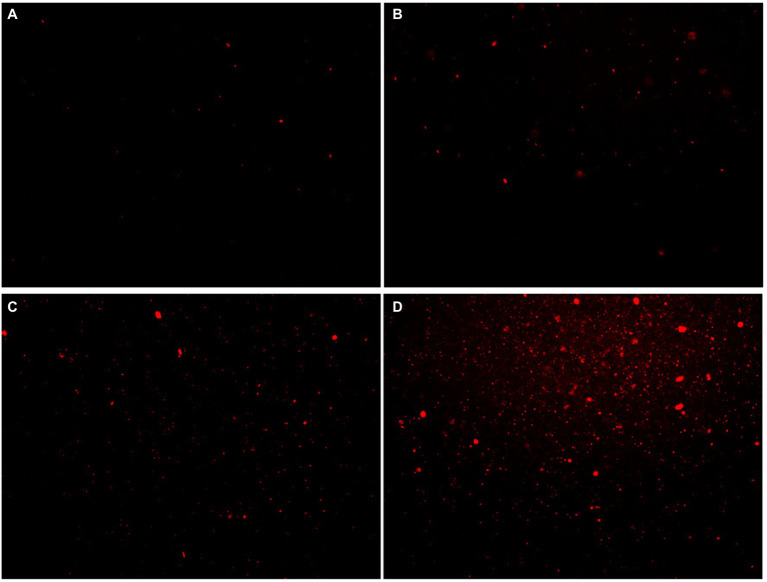
Effects of moringin on cell membrane integrity of *Listeria monocytogenes* by inverted fluorescence microscope. **(A)** Untreated *Listeria monocytogenes*. **(B)** *Listeria monocytogenes* treated with moringin at 1/2 MIC. **(C)** *Listeria monocytogenes* treated with moringin at 1 MIC. **(D)** *Listeria monocytogenes* treated with moringin at 2 MIC.

Propidium iodide (PI) dye can only penetrate the damaged cells and bind to nucleic acids, resulting in increased fluorescence intensity. The effect of moringin on the cell membrane integrity of *L. monocytogenes* was investigated by a fluorescence probe (Figure 5). The results showed that the fluorescence was enhanced with increasing chorismate concentration. This result indicated that 1/2 MIC, 1 MIC, and 2 MIC concentrations of moringin induced cell membrane damage, reduced cell membrane integrity, and increased cell membrane permeability. In addition, with increasing moringin concentration, the cell membrane damage of *L. monocytogenes* was enhanced.

#### Moringin Causes Oxidative Stress in *Listeria monocytogenes*

**Figure 6:**
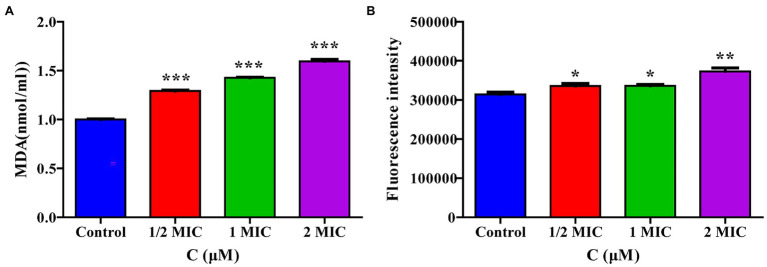
Effect of moringin on oxidative stress apoptosis in *Listeria monocytogenes*. **(A)** MDA level activity exposure to different concentrations of moringin. **(B)** ROS activity exposure to different concentrations of moringin. *p < 0.05, **p < 0.01, and ***p < 0.001.

The transcriptome sequencing results suggested that moringin may induce oxidative stress in cells. The intracellular MDA contents and ROS activity were evaluated in this study. The MDA content results are shown in Figure 6A. After treatment with 1/2 MIC, 1 MIC, or 2 MIC concentrations of moringin, the MDA levels were significantly increased due to oxidative stress injury (*p* < 0.001). Furthermore, in comparison with those of the control group, the ROS levels in cells were significantly higher after treatment with 1/2 MIC, 1 MIC, and 2 MIC concentrations of moringin (Figure 6B).

#### Moringin Interferes With the Replication of *Listeria monocytogenes* DNA

**Figure 7:**
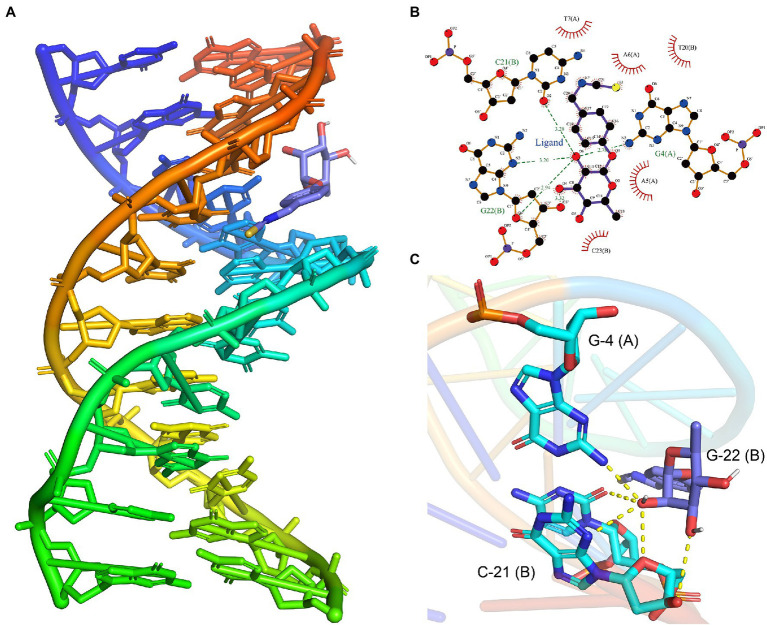
Effects of moringin on DNA of *Listeria monocytogenes* by molecular docking. **(A)** Binding poses between moringin and DNA. **(B)** The 2D plot of the interactions between moringin and DNA. **(C)** The 3D plot of the interactions, with different base names marked. The letters in parentheses represented different strands of DNA.

The above transcriptome sequencing results suggested that DNA binding-related genes were changed after the treatment of *L. monocytogenes* with moringin. Therefore, molecular mock docking of moringin and DNA was performed. DNA and moringin were relatively easily bound together, which showed that moringin fit into the grooves in the DNA (Figure 7A). Interaction analysis showed that moringin formed hydrogen bonds with DNA bases (Figure 7B). O6 of moringin formed a 2.78 Å hydrogen bond with G4 (A), a 3.28 Å hydrogen bond with C21 (B), and two hydrogen bonds with G22 (B), with lengths of 3.20 Å and 2.94 Å, respectively. Additionally, some hydrophobic interactions were observed between moringin and A5 (A), A6 (A), T7 (A), T20 (B), and C23 (B). The 3D visualization of the complex is shown in Figure 7C. Both the interaction analysis and the free energy calculation indicated that the binding between DNA and moringin was stable. As with the transcriptome analysis, moringin may insert into the DNA base pairs of *L. monocytogenes* and impede DNA replication.

#### Moringin Interfered With the Energy Metabolism of *Listeria monocytogenes*

**Figure 8:**
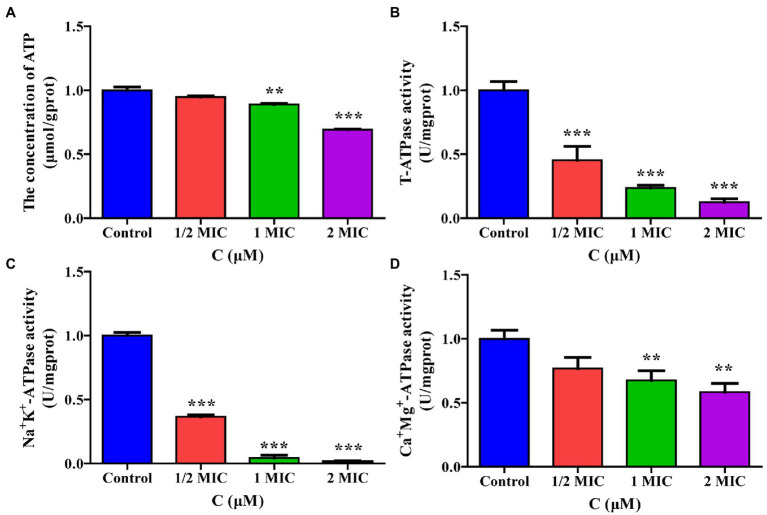
Effect of moringin on energy metabolism in *Listeria monocytogenes*. **(A)** ATP levels exposure to different concentrations of moringin. **(B)** T-ATPase activity exposure to different concentrations of moringin. **(C)** Na^+^K^+^-ATPase activity exposure to different concentrations of moringin. **(D)** Ca^2+^Mg^2+^-ATPase activity exposure to different concentrations of moringin. *p < 0.05, **p < 0.01, and ***p < 0.001.

To verify the effect of moringin treatment on energy metabolism in *L. monocytogenes*, the intracellular ATP content and ATPase activity were measured. As shown in Figure 8A, the intracellular ATP concentrations in *L. monocytogenes* decreased significantly after treatment with 1 MIC and 2 MIC concentrations of moringin, compared with those of the control group.

The effect of moringin on the activities of total ATPase (T-ATPase), Na^+^K^+^-ATPase, and Ca^+^Mg^+^-ATPase of *L. monocytogenes* are shown in Figures 8B–D. The activities of T-ATPase and Na^+^K^+^-ATPase in *L. monocytogenes* were significantly decreased after treatment with 1/2 MIC, 1 MIC, and 2 MIC concentrations of moringin compared to those of the control group (*p* < 0.001; Figures 8B,C). Additionally, the Ca^+^Mg^+^-ATPase activity of *L. monocytogenes* was significantly decreased after treatment with 1 MIC and 2 MIC concentrations of moringin y (*p* < 0.01; Figure 8D).

## Discussion

Moringin results from the myrosinase hydrolysis of glucomoringin, the most abundant glucosinolate in *Moringa oleifera* seeds (Rajan et al., 2016). In this study, the antibacterial activity of moringin against the food-borne pathogen *L. monocytogenes* was investigated, and its mechanism was elucidated at the molecular level for the first time. We found that moringin inhibits the growth of *L. monocytogenes* by disrupting cell wall and cell membrane structure, stimulating oxidative stress, interfering with energy metabolism and DNA replication, and ultimately leading to its death. Understanding moringin’s antimicrobial action route will aid in its use in the food sector and boost the added value of *M. oleifera* seeds, which is critical for regional economic development.

In the present study, the MIC of moringin against *L. monocytogenes* was 400 μM (0.124 mg/ml; Table 2). Compared to that of phenyllactic acid (1.25 mg/ml; Ning et al., 2017) and the lectin from pomegranate sarcotesta (0.0125 mg/ml; Silva et al., 2021), moringin showed a moderate inhibitory effect against *L. monocytogenes*. Moringin is one of the isothiocyanates. Due to the structure of the carbon chains of isothiocyanates, they tend to have different MICs. For example, the MICs of phenylethyl isothiocyanate (PEITC) against Shiga toxin-producing and enterotoxigenic *Escherichia coli* were both 400 μmol/L (Yang et al., 2020). Other research has reported that the MIC of sulforaphene against *Helicobacter pylori* was 600 mg/ml (Lim et al., 2016). The strong antibacterial activity of moringin may be attributed to the fact that it is a isothiocyanate, like benzyl isothiocyanate (BITC) and phenylethyl isothiocyanate (PEITC).

The transcriptome enables us to examine the whole response simultaneously and globally at the transcriptional level. Transcriptome technology has become a vital tool in the biological sciences (Masri et al., 2021). RNA-seq has sped research to better understand the complexities of gene expression, regulation, and networks (Daniel et al., 2015). We employed transcriptomic approaches to obtain a better understanding of the possible connections between moringin and *L. monocytogenes*. As demonstrated by comparing RNA-seq data from the experimental and control groups, moringin affects the integrity of cell walls and cell membranes, induces oxidative stress, and impairs energy metabolism and DNA replication. These results were subsequently verified through a validation of the antibacterial mechanism.

Peptidoglycan is the major component of bacterial cell walls and plays a crucial role in maintaining cell wall integrity and bacterial morphology (Typas et al., 2011). Peptidoglycan is considered a specific target for many drugs (Typas et al., 2011). In the present study, the transcriptomic results showed the MurA and MurC genes, which are associated with peptidoglycan biosynthesis, were significantly downregulated in the moringin-treated group (Supplementary Figure S1). As with the transcriptome results, SEM revealed that moringin treatment inhibited *L. monocytogenes* by disrupting the structure of the cell wall (Figure 4). ABC transporter proteins are a group of biological transport proteins with a wide range of functions in the plasma membrane of microorganisms. They are mainly responsible for the ATP-driven transport of various substrates across the membrane (Kafantaris et al., 2021). In this study, the ABC transporter was also regulated (Supplementary Figure S2). Spermidine and putrescine are important polyamines. Alterations in the transport capacity of polyamines may affect cell proliferation and differentiation (Kashiwagi et al., 1995, 1996). The PotA is a membrane-associated protein, and PotD is a periplasmic substrate-binding protein. The PotA and PotD genes are involved in the spermidine/putrescine transport system. Their expression levels were both upregulated in this study. In addition, genes for osmoprotectant, such as opuA, opuB, and opuC, were downregulated, suggesting that cells are affected by osmotic stress (Ko et al., 1994; Ko and Smith, 1999). The influxes of PI indicated that moringin damaged the membrane, decreasing its integrity and increasing its permeability (Figure 5). Cell wall and cell membrane have been extensively studied as antimicrobial targets. Once the structure of the cell wall and cell membrane is damaged, it leads to leakage of nucleic acids and proteins from the cell, causing irreversible damage and eventually cell death. The present study suggests that Moringin damaged the integrity cell wall of *L. monocytogenes*, and increased the permeability of the cell membrane. In agreement with our study, the study by Zhao et al. (2020a) also found significant changes in the permeability of the cell membrane of *L. monocytogenes* with increased nucleic acid and protein leakage when nisin and grape seed extract were combined.

Cell motility plays an essential role in host–microbe interactions. The colonization and virulence properties of bacteria include several biological processes, including signal transduction, chemotaxis, and flagellar motility (Vasconcelos et al., 2018). Flagellar motility and chemotaxis are two critical features of bacterial attachment that enable them to survive in various environments (Schurch et al., 2015). The flagellum comprises the basal part, the flagellar hook, and the flagellar filament. The basal body, which is embedded in the cell wall and cytoplasmic membrane, acts as a molecular motor; the flagellar hook, which connects the motor spindle to the flagellar filament, acts as a torque transmitter; and the filament, which is driven by the motor, rotates and generates propulsive force to drive the bacteria (Tu et al., 2020). In this study, treatment with moringin resulted in significant upregulation of the FliH and FliI genes (Supplementary Figure S3), indicating that moringin may have inhibited the function of the flagellar energy export system of *L. monocytogenes* to some extent through the upregulation of these two genes. In turn, the FlgL and FlgK genes of flagellar hooks were also upregulated (Supplementary Figure S3), leading to an increase in bacterial motility. Interestingly, the genes CheA and CheY genes, which are associated with chemotaxis, were also significantly upregulated in *L. monocytogenes* after moringin treatment (Supplementary Figure S4). It is possible that *L. monocytogenes* transmits stimulus signals from the external environment to flagellin to respond to chemotaxis accordingly.

The phosphotransferase system (PTS) involves the uptake and phosphorylation of carbohydrates from the extracellular environment (Kotrba et al., 2001). In this study, the genes MltA, GatA, GatB, and GatC, which are related to the phosphotransferase system, were significantly upregulated after treatment with moringin, indicating an increase in carbohydrate uptake, which may be a self-protective strategy of *L. monocytogenes* to maintain carbohydrate metabolism for cellular energy supply under moringin stress (Supplementary Figure S5).

The oxidative stress response of pathogens to external stimuli is generally used to understand their acclimatization mechanisms (Maurya et al., 2020). In the present study, intracellular ROS and MDA production were increased (Figure 6), verifying that ROS and MDA-mediated oxidative stress played a significant role in the antimicrobial effect of moringin against *L. monocytogenes*. Previous studies have found that the generation of ROS could lead to DNA damage (Salehi et al., 2018). DNA is one of the most important genetic materials, and its disruption could hamper gene expression, thereby leading to the blocking of normal enzyme and receptor synthesis and further causing the death of bacteria (Li et al., 2019a). In this study, GO analysis showed that moringin could affect the DNA binding (Figure 2C). Thus, we further investigated the interaction of moringin with DNA by molecular docking (Figure 7). The results suggested that moringin could probably bind to DNA and lead to changes in DNA conformation and structure. Therefore, the results of this study indicated that DNA might be another antibacterial target of moringin, which enriched the knowledge of the antibacterial mechanism of moringin.

The energy balance in organisms can be severely affected by environmental stress, which requires additional energy to maintain or restore dynamic equilibrium (Wang et al., 2018). In most organisms, the tricarboxylic acid cycle (TCA cycle) is a vital pathway for energy production (Korotetskiy et al., 2020). In this study, moringin downregulated the expression of all genes associated with the TCA cycle (Supplementary Figure S6) and upregulated most genes related to fructose, mannose, and propionate metabolism (Supplementary Figures S7, S8). ATP plays a key role in cellular energy metabolism. Our results demonstrated that the ATPase activity and the ATP content of cyanobacteria decreased significantly after treatment with moringin (Figure 8). Therefore, moringin may interfere with energy metabolism by affecting the carbohydrate metabolism of *L. monocytogenes*, further affecting the use of its catabolic products for cellular repair and thus inhibiting bacterial growth.

This research studied the antimicrobial effect and its potential mechanism of moringin against *L. monocytogenes*. *Listeria monocytogenes* is a highly dangerous bacterial pathogen that can cause a severe and often fatal disease, once persons are infected by it. *Listeria innocua* is an innocuous subspecies of *Listeria* with high morphological, serological, and biochemical characteristics similar to *L. monocytogenes* (Chen et al., 2019). Therefore, for safety reasons, *L. innocua* can be used as a suitable alternative for food safety research.

## Conclusion

In summary, this study evaluated the inhibitory activity and mechanism of moringin against the food-borne pathogen *L. monocytogenes*. It was found that moringin inhibited the growth of *L. monocytogenes* in a dose- and time-dependent manner. The transcriptome results suggested that moringin significantly affected genes related to the cell wall and membrane, oxidative stress, energy metabolism, cell motility, and DNA binding. We further verified that, as with the transcriptomic results, moringin inhibited *L. monocytogenes* by disrupting the cell wall and membrane structure and composition, inducing oxidative stress, interfering with energy metabolism, affecting cell motility, and preventing DNA replication.

## Data Availability Statement

The datasets presented in this study can be found in online repositories. The names of the repository/repositories and accession number(s) can be found in the article/**Supplementary Material**.

## Author Contributions

YW: conceptualization, investigation, writing—original draft, and project administration. WL: investigation, methodology, and validation. RS, MY, XL, and NZ: validation. LL: conceptualization, supervision, writing—review and editing, and project administration. JS and YT: conceptualization, supervision, and project administration. All authors contributed to the article and approved the submitted version.

## Funding

This work was supported by the “Major Project of Science and Technology Department of Yunnan Province” (2018ZI001 and 202002AA100005), “YEFICRC project of Yunnan provincial key programs” (2019ZG009), and “Yunnan Province Young and Middle-aged Academic and Technical Leaders Reserve Talents Project” (2018HB040).

## Conflict of Interest

The authors declare that the research was conducted in the absence of any commercial or financial relationships that could be construed as a potential conflict of interest.

## Publisher’s Note

All claims expressed in this article are solely those of the authors and do not necessarily represent those of their affiliated organizations, or those of the publisher, the editors and the reviewers. Any product that may be evaluated in this article, or claim that may be made by its manufacturer, is not guaranteed or endorsed by the publisher.

## Footnotes

^1^ www.majorbio.com

^2^ http://www.rcsb.org

^3^ https://pubchem.ncbi.nlm.nih.gov/compound/153557

^4^ http://www.pymol.org/
