# Supplementary material for: Multi-Target Antibacterial Mechanism of Moringin From Moringa oleifera Seeds Against Listeria monocytogenes
Source: Front Microbiol. 2022 Jun 8;13:925291. doi: 10.3389/fmicb.2022.925291 (PMC9213813; doi:10.3389/fmicb.2022.925291)

Supplementary Material

# Supplementary Figures

**Supplementary Figure S1** Peptidoglycan biosynthesis pathway from the KEGG annotation analysis. The genes with red/blue borders belong to the differential genes detected by RNA- sequencing, in which red represents the upregulated genes, and blue represents the down-regulated genes.

**Supplementary Figure S2** ABC transporter pathway from the KEGG annotation analysis. The genes with red/blue borders belong to the differential genes detected by RNA- sequencing, in which red represents the upregulated genes, and blue represents the down-regulated genes.

**Supplementary Figure S3** Flagellar assembly pathway from the KEGG annotation analysis. The genes with red/blue borders belong to the differential genes detected by RNA- sequencing, in which red represents the upregulated genes, and blue represents the down-regulated genes.

**Supplementary Figure S4** Bacterial chemotaxis pathway from the KEGG annotation analysis. The genes with red/blue borders belong to the differential genes detected by RNA- sequencing, in which red represents the upregulated genes, and blue represents the down-regulated genes.

**Supplementary Figure S5** Phosphotransferase system (PTS) pathway from the KEGG annotation analysis. The genes with red/blue borders belong to the differential genes detected by RNA- sequencing, in which red represents the upregulated genes, and blue represents the down-regulated genes.

**Supplementary Figure S6** Tricarboxylic acid cycle (TCA cycle) pathway from the KEGG annotation analysis. The genes with red/blue borders belong to the differential genes detected by RNA- sequencing, in which red represents the upregulated genes, and blue represents the down-regulated genes.

**Supplementary Figure S7** Fructose and mannose metabolism pathway from the KEGG annotation analysis. The genes with red/blue borders belong to the differential genes detected by RNA- sequencing, in which red represents the upregulated genes, and blue represents the down-regulated genes.

**Supplementary Figure S8** Propanoate metabolism pathway from the KEGG annotation analysis. The genes with red/blue borders belong to the differential genes detected by RNA- sequencing, in which red represents the upregulated genes, and blue represents the down-regulated genes.

**Supplementary Figure S1**


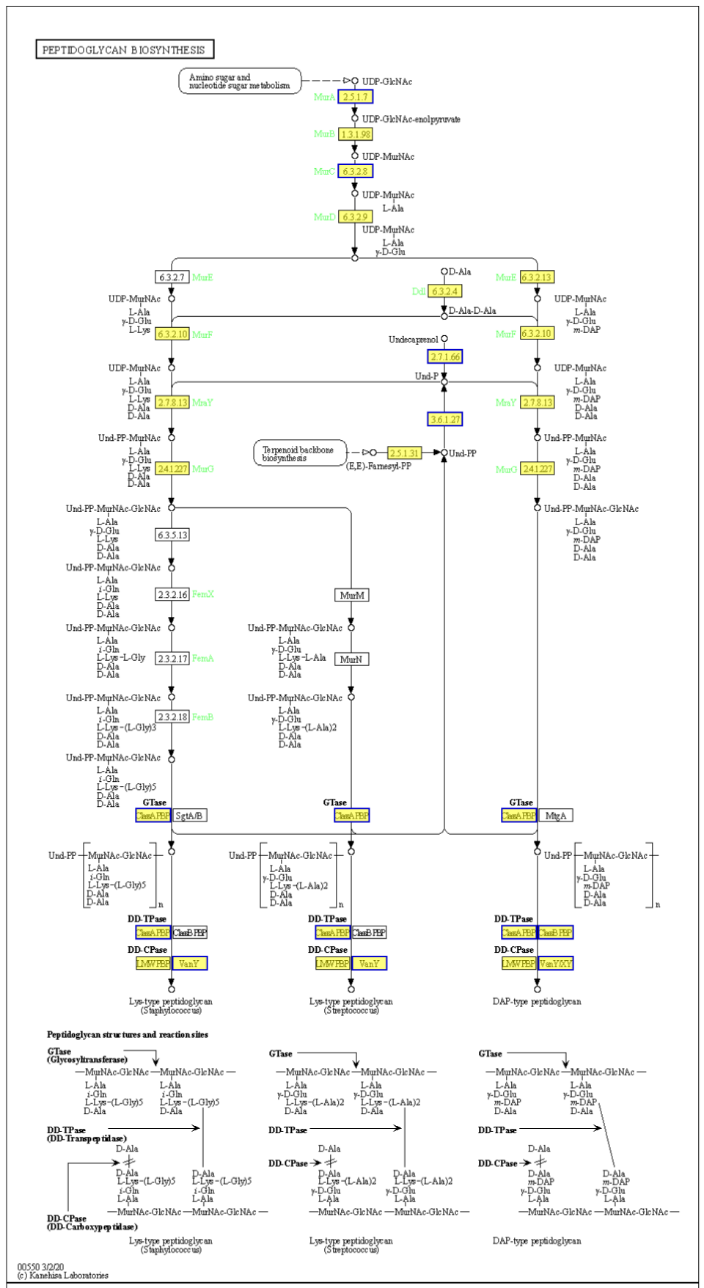


**Supplementary Figure S2**


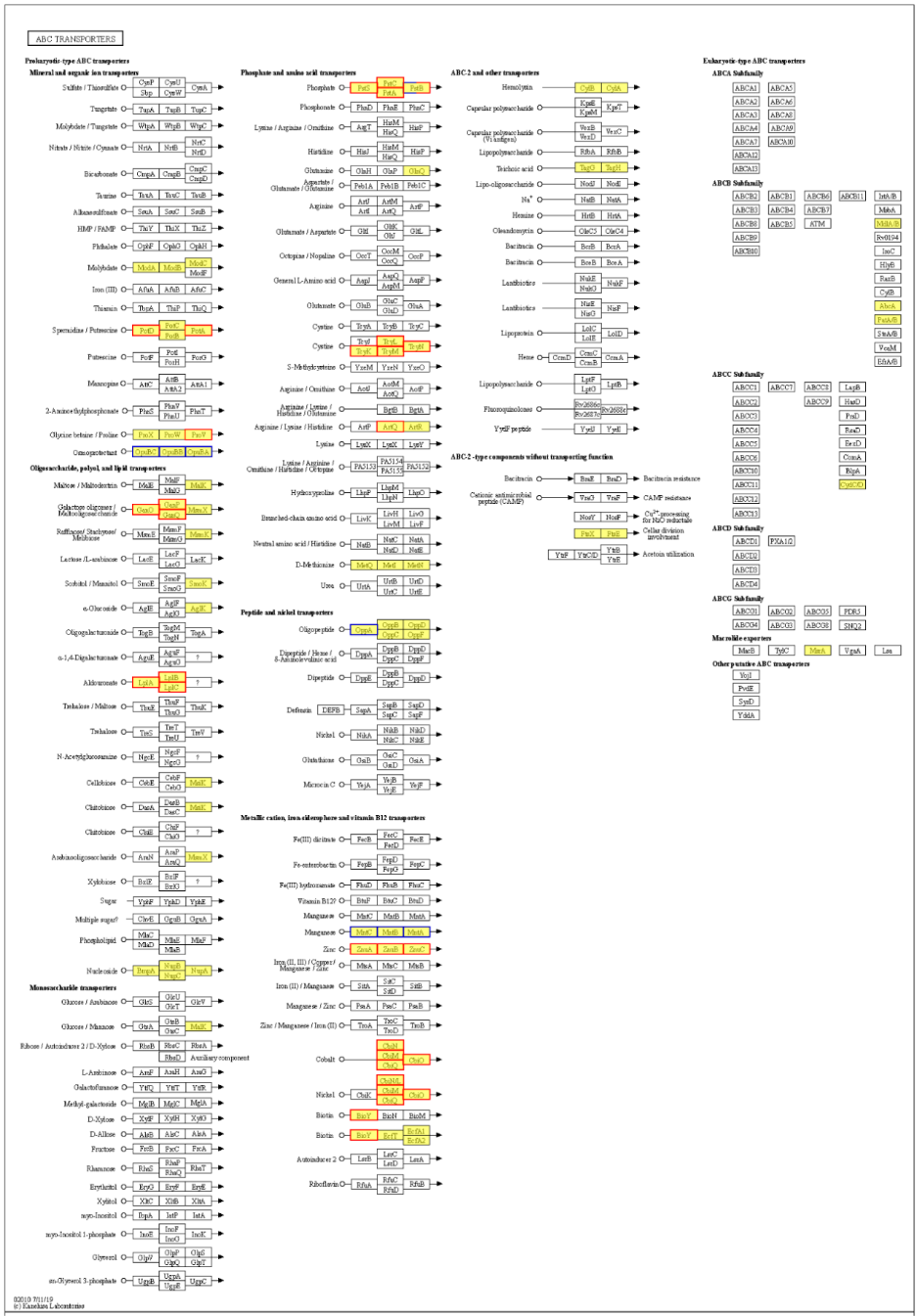


**Supplementary Figure S3**


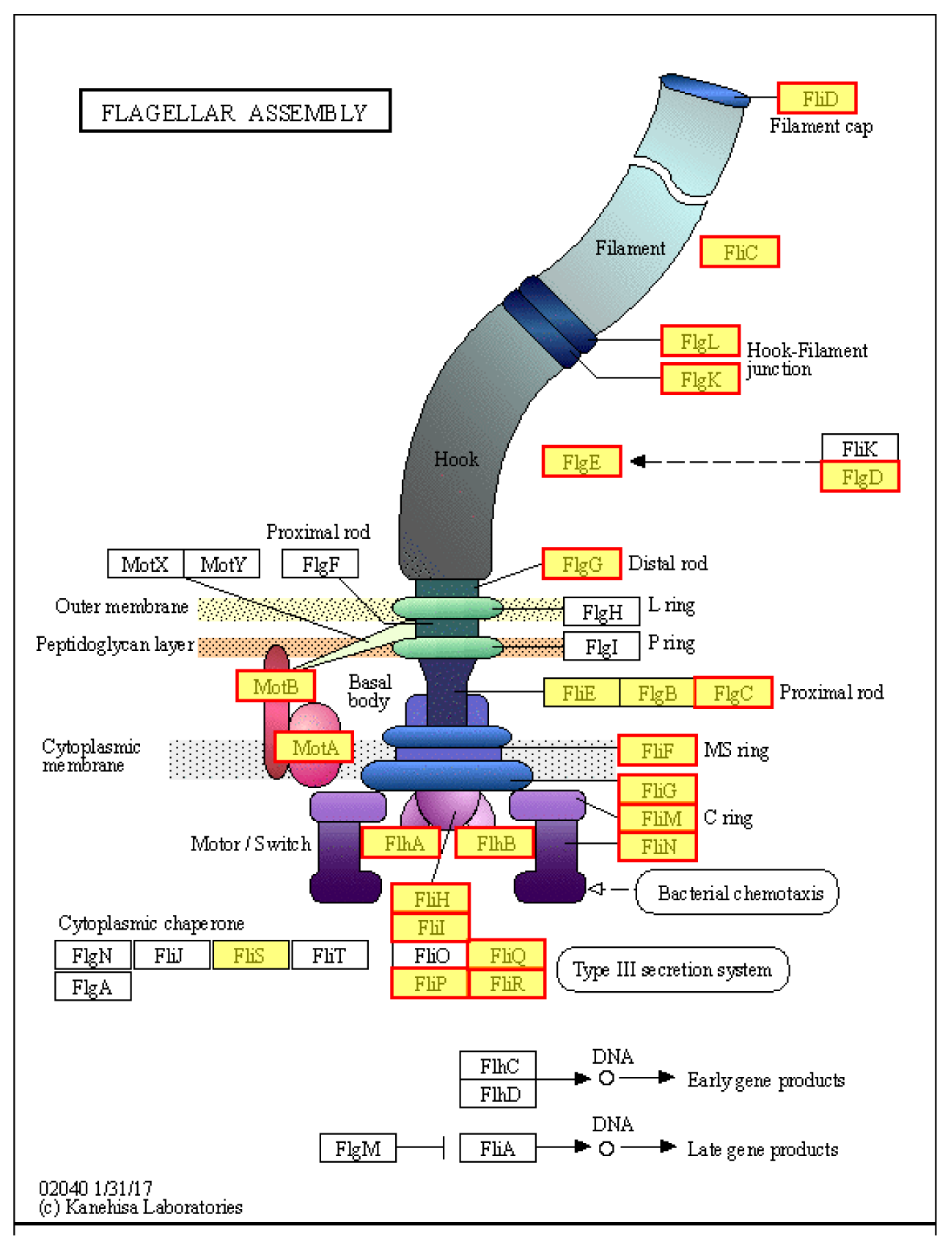


**Supplementary Figure S4**


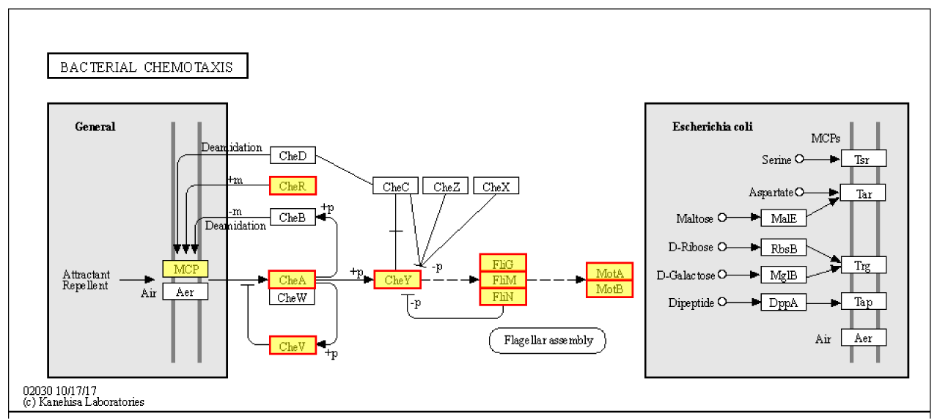


**Supplementary Figure S5**


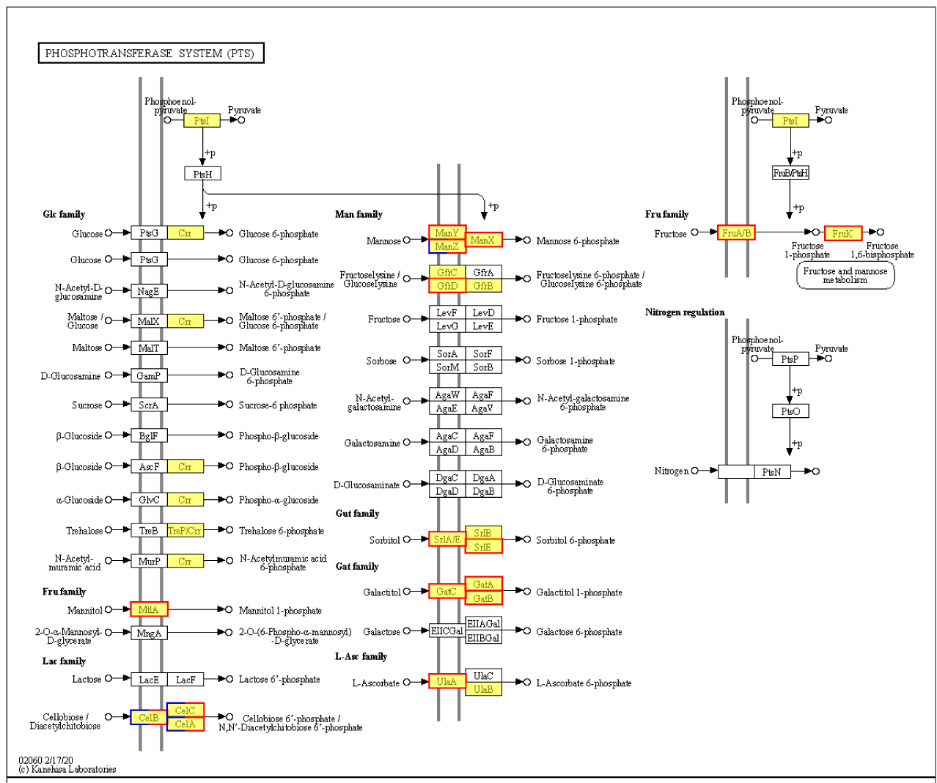


**Supplementary Figure S6**


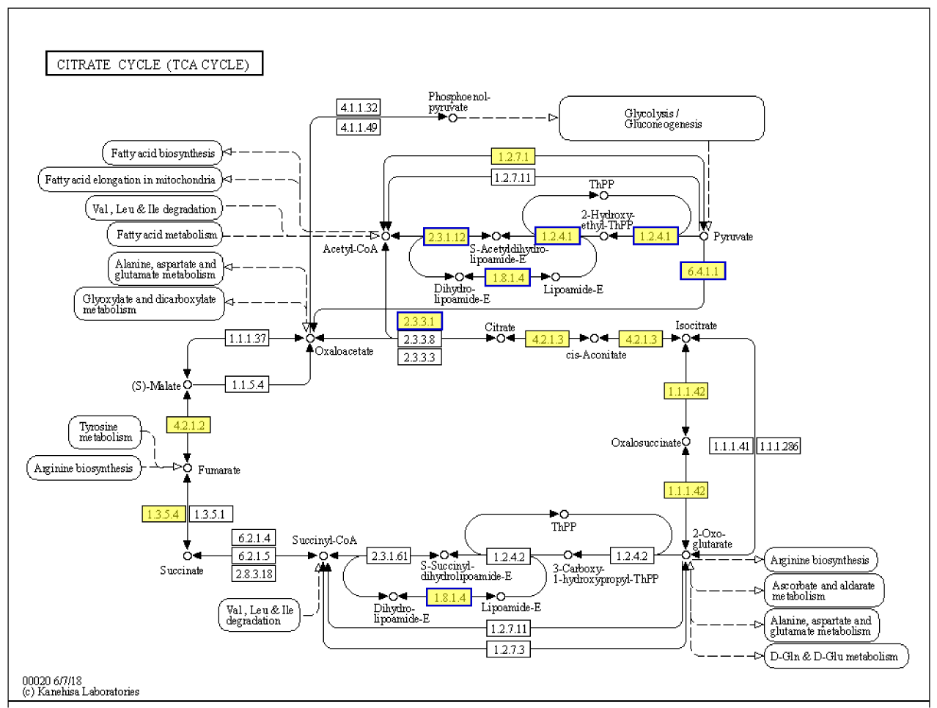


**Supplementary Figure S7**


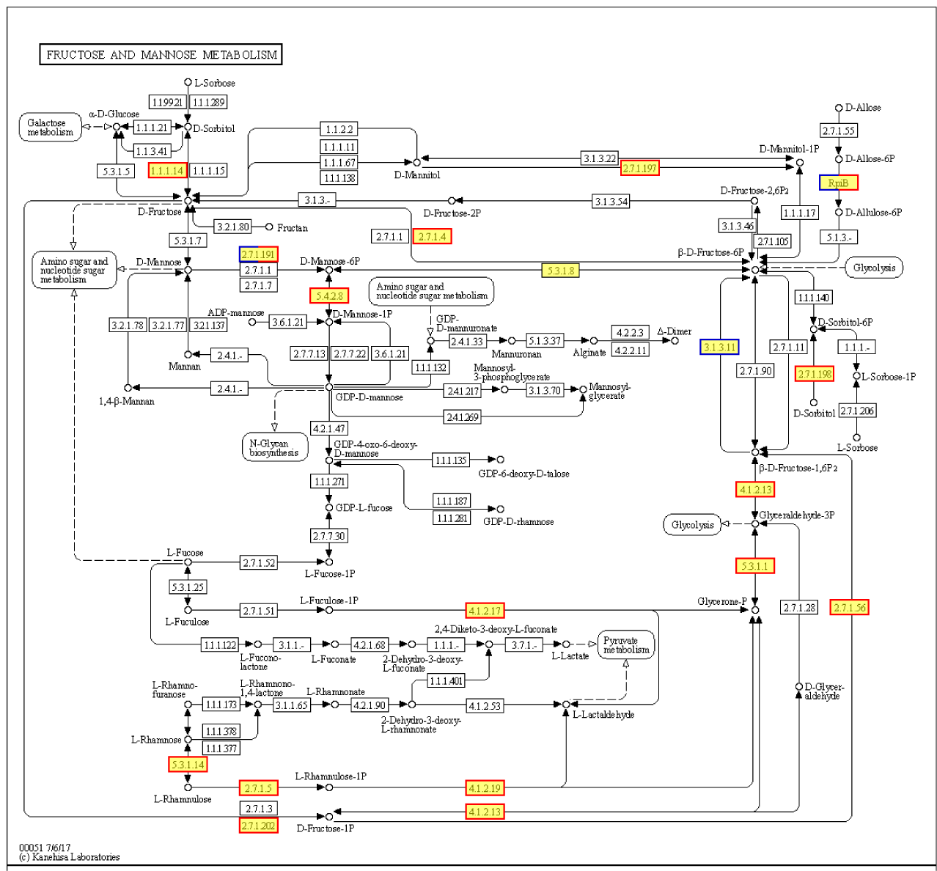


**Supplementary Figure S8**


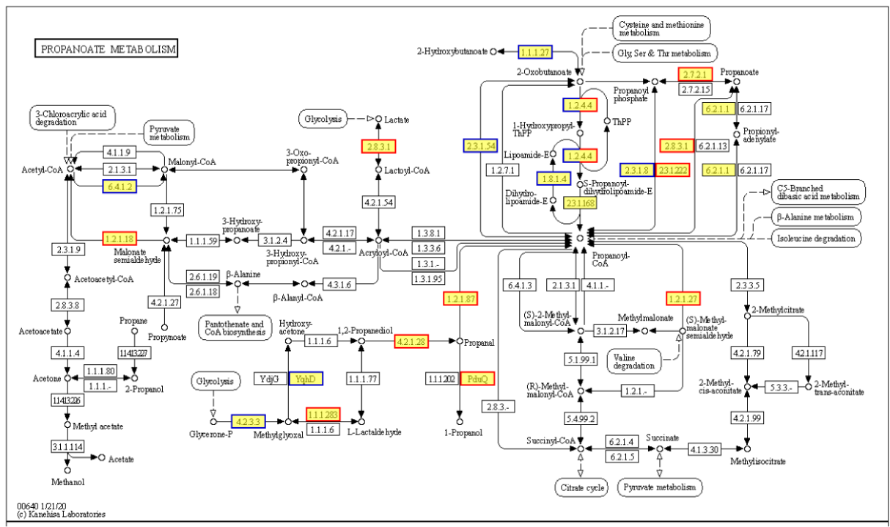

Supplement: Supplementary file 1 [file Data_Sheet_1.docx]
